# On the Use of Quinine

**Published:** 1847-02

**Authors:** A. W. Benton

**Affiliations:** Sterling, Whitesides co., Ill.


ARTICLE III.

*On the use of Quinine.* By A. W. Benton, M.D., of Sterling, Whitesides co., Ill.

What is the condition of the system, and what the indications which require the use of quinine?

As much as has of late been written upon the use of quinine, the answer to the above question seem still to be a desideratum in the practice of medicine. The science of medicine can be improved only by a long and careful observation of facts and phenomena as they occur around us.

These facts have to be collected, compared, and their different relations and bearings carefully noted, before any definite law, or rule of action, can be established.

As these facts and phenomena are not confined to the observation of a favored few, but are spread abroad upon the open page of nature, it often happens that a man in the humbler walks of life may discover some important truth, which the far directed ken of a more eminent man may have overlooked. Or, if he is not so fortunate as to make any new discovery, he may at least observe some trifling circumstance, that may go far to establish the truth of some previous discovery.

This being the case, it is the duty of every physician to be vigilant at his post, however limited the sphere of his observation.

It seems to me that some are too indiscriminate in the use of quinine, while others are too cautious. That quinine has a decided and specific action upon the system, under certain circumstances, is a well established fact.

This being the case it must be adapted to some peculiar state of the system, indicated by a certain train of symptoms. What this state of the system is, and what are its symptoms, have been subjects of study and observation with me for the last seven years, the length of time I have resided in Illinois.

The result of my observations is, that an impoverished and morbid state of the blood, causing a diminution of the contractility of the heart and arteries, and functional action of the capillary vessels, constitute that state of the system which calls for the use of quinine. And that this state is indicated by a more or less well marked tendency to exacerbation and

*remission*, if not intermission: and never characterized by that hard, unyielding, and bounding pulse which attends acute inflammation.

In the height of a paroxysm of fever, the pulse may approximate to the character of an inflammatory pulse, but still I have always found it to lack that hardness which it assumes in inflammation. The coats of the artery do not feel so rigid, and tense, and unyielding. The action of the heart and arteries seems to be more of a tumultuous action, as if it proceeded from irritation, as I believe it does; and *that* irritation caused by an accumulation of blood in the large vessels; and this accumulation, in its turn, produced by the previous diminished action which proved insufficient to carry on the natural circulation through the capillary system of vessels; which are, at the same time, laboring under, or rather ceasing to labor under, a diminished supply of nervous energy or influence.

The true theory of intermittent fever seems to me to be something like the following.

A vitiated state of the atmosphere, consisting probably in the admixture of some exhaled gaseous matter, together with a changed electrical condition, imparts to the blood an unhealthy, or morbid quality. This morbid blood, together with the electrical change, produces a diminished energy in that portion of the nervous system which presides over the circulating and organic functions of the body.

The consequence is, the blood does not stimulate the heart and arteries sufficiently to keep up their accustomed contractility, and elasticity. The capillaries at the same time are incapacitated for the performance of the functions assigned them.

From all this results a diminished action of the heart, arteries, and capillaries, insufficient to carry on the circuit of the circulation—the blood ceases gradually to find its way into the small vessels, and gradually accumulates in the large vessels.

When the capillaries get sufficiently empty, the consequence is a rigor or chill. When the large vessels get sufficiently full, or full for a sufficient length of time, the consequence is irritation and reaction, which forces the circuit of circulation till an equilibrium is established; then the reaction ceases, leaving the system in comparative health.

Then again, during the intermission, commences and continues the same diminished action, and gradual accumulation of blood in the larger vessels, and emptiness of the capillaries, till the same phenomena, in a longer or shorter period, are again produced; thus accounting for the periodicity of agues.

The action of quinine I conceive to be to neutralize the sedative poison of malaria, and restore to the blood its appropriate stimulus for calling into action the heart, arteries and capillaries, giving them tone, vigor, and stability of action.

